# Generation of human chemically induced pluripotent stem cells from UC-MSCs

**DOI:** 10.1186/s13619-026-00295-9

**Published:** 2026-07-01

**Authors:** Guangqiang Gong, Shanshan Wen, Siyi Huang, Ran Liu, Ran Zheng, Wei Jiang

**Affiliations:** 1 Department of Biological Repositories, Frontier Science Center for Immunology and Metabolism, Medical Research Institute, Zhongnan Hospital of Wuhan University, Wuhan University, Wuhan, 430071 China; 2 The Institute of Translational Medicine and Jiangxi Province Key Laboratory of Precision Cell Therapy, The Second Affiliated Hospital, School of Basic Medical Sciences and Institute of Biomedical Innovation, Jiangxi Medical College, Nanchang University, Nanchang, 330031 China; 3 Hubei Provincial Key Laboratory of Developmentally Originated Disease, Wuhan, 430071 China; 4 Shenzhen Beike Biotechnology Co., Ltd, Shenzhen, 518063 China

Dear Editor,

Reprogramming technology can convert somatic cells into pluripotent stem cells (PSCs) with self-renewal and multi-lineage differentiation potential, which breaks the inherent boundaries of cellular identity and holds great promise for regenerative medicine and cell replacement therapy (Wen et al. 2025). Transcription factor-based reprogramming overcomes the technical difficulties and ethical issues that somatic cell nuclear transfer faces, but its safety due to massive genetic manipulations remains a challenge for clinical application (Hui & Yamanaka 2024). Chemical reprogramming, which uses a small-molecule compound cocktail to manipulate cell fate, offers greater safety due to its non-integrating nature and easier process control, opening a new path for clinical-grade cell production (Wen et al. 2025). In 2022, Deng’s group successfully established the chemical reprogramming approach to convert human somatic adipose-derived stromal cells (ADSCs) into chemically induced pluripotent stem cells (CiPSCs) (Guan et al. 2022). Later, the group further established a three-stage chemical reprogramming protocol, significantly increasing the efficiency and robustness of ADSC reprogramming (Liuyang et al. 2023). Recently, by inhibiting histone acetyltransferases to trigger swift epigenetic switching, Deng’s group effectively overcame ADSC donor variability and significantly shortened the reprogramming timeframe (Wang et al. 2025).

Although human CiPSCs have been generated from somatic cells, the reported applicable starting cell types remain limited. The first success was achieved by human ADSCs and dermal fibroblasts (Guan et al. 2022), and very recently, an adjusted protocol was reported to work with blood cells (Peng et al. 2025; Wang et al. 2026). Notably, these protocols rely on hypoxic culture conditions (5% O₂), which require specialized equipment and procedures, thereby increasing operational complexity. Differing significantly from previous protocols for ADSCs and dermal fibroblasts, the newly developed protocol centered on screening small molecules to eliminate blood cell identity and drive the critical transition from suspension to adherent states for reprogramming. Therefore, the universality of chemical reprogramming protocols across different cell types still requires systematic evaluation and optimization.

Here, we first repeated the reported original three-stage protocol (Liuyang et al. 2023) to induce CiPSCs from human ADSCs derived from one breast adipose tissue sample (Fig. S1A). Although OCT4-positive cells were observed in Stage 3, they were present in very low numbers and did not form compact colonies (Fig. S1B), subsequently dying during attempts to establish a stable cell line. Given that Stage 1, which involves erasuring somatic cell identity, was considered the most critical step, we later focused on modifying Stage 1. First, we titrated the concentrations of seven key small molecules and found that increasing the concentration of most molecules promoted the expression of *LIN28A* in our experiments (Fig. 1A, S1C). Second, we tested different serum-replacement reagents and found that either 1% ITS-X or increased B27 (4% B27) could significantly promote cell proliferation, transitioning the cells to an epithelial-like morphology, and significantly enhancing *LIN28A* expression (Fig. 1B, S1D). In addition, to improve the economy and convenience of chemical reprogramming, we selected the small-molecule hypoxia mimetic deferoxamine mesylate (DFOM) under normoxic conditions and found that 4 μM DFOM effectively increased *LIN28A* expression (Fig. 1C, S1E).

**Fig. 1:**
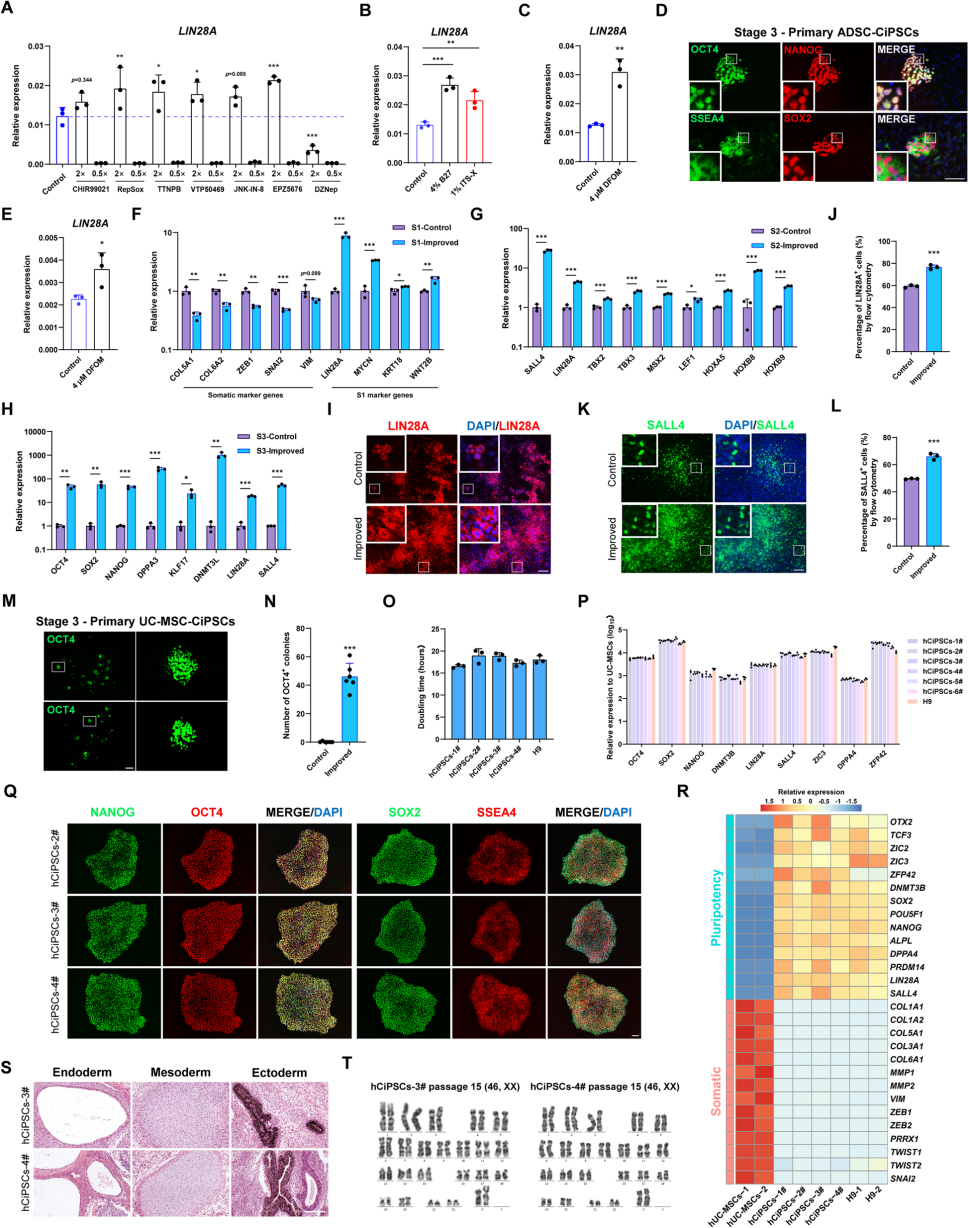
Generation and characterization of CiPSCs from ADSCs and UC-MSCs. **A** RT-qPCR analysis of *LIN28A* at the end of Stage 1 of ADSC reprogramming upon concentration titrations of core small molecules under the original protocol (control). Data represented as mean ± SD (*n* = 3). **B** RT-qPCR analysis of *LIN28A* at the end of Stage 1 of ADSC reprogramming following supplementation with 4% B27 or 1% ITS-X under the original protocol (control). Data represented as mean ± SD (*n* = 3). **C** RT-qPCR analysis of *LIN28A* at the end of Stage 1 of ADSC reprogramming following supplementation with 4 μM DFOM under the original protocol (control). Data represented as mean ± SD (*n* = 3). **D** Immunofluorescence of pluripotency marker genes in primary CiPSCs from ADSCs at the end of Stage 3 under the modified protocol. Scale bar, 100 μm. **E** RT-qPCR analysis of *LIN28A* at the end of Stage 1 of UC-MSC reprogramming following supplementation with 4 μM DFOM under the original protocol (control). Data represented as mean ± SD (*n* = 3). **F-H** RT-qPCR analysis of somatic and first-stage marker genes at the end of Stage 1 (**F**), second-stage marker genes at the end of Stage 2 (**G**) and pluripotency marker genes at the end of Stage 3 (**H**) of UC-MSC reprogramming under the original protocol (control) and improved protocol. Data represented as mean ± SD (*n* = 3). **I** Immunofluorescence of LIN28A at the end of Stage 1 of UC-MSC reprogramming under the original protocol (control) and improved protocol. Scale bar, 100 μm. **J** Flow cytometric analysis of LIN28A at the end of Stage 1 of UC-MSC reprogramming under the original protocol (control) and improved protocol. Data represented as mean ± SD (*n* = 3). **K** Immunofluorescence of SALL4 at the end of Stage 2 of UC-MSC reprogramming under the original protocol (control) and improved protocol. Scale bar, 100 μm. **L** Flow cytometry analysis of SALL4 at the end of Stage 2 of UC-MSC reprogramming under the original protocol (control) and improved protocol. Data represented as mean ± SD (*n* = 3). **M** OCT4 staining at the end of Stage 3 of UC-MSC reprogramming under the improved protocol. Scale bar, 300 μm. **N** Number of CiPSC colonies from UC-MSCs at the end of Stage 3 under the original protocol (control) and improved protocol. Data represented as mean ± SD (*n* = 6, one well of a 24-well plate was counted as one group). **O** Calculated doubling times for CiPSCs from UC-MSCs and ESC H9. Data represented as mean ± SD (*n* = 3). **P** RT-qPCR analysis of pluripotency marker genes in CiPSCs from UC-MSCs. ESC H9 was used as a reference. Data represented as mean ± SD (*n* = 3). **Q** Immunofluorescence of pluripotency markers in CiPSCs from UC-MSCs. Scale bar, 100 μm. **R** Heatmaps showing the expression of pluripotency- and somatic-related genes in UC-MSCs, CiPSCs, and ESC H9. **S** Hematoxylin and eosin (H&E) staining of teratoma sections from UC-MSC-derived CiPSCs. For each CiPSC line, images contained tissues representative of endoderm, mesoderm, and ectoderm from the same teratoma. Scale bar, 100 μm. **T** Karyotype analysis showing a normal diploid chromosome content in CiPSCs from UC-MSCs

Combined with such modifications, the expression levels of *LIN28A* at the end of Stage 1 and pluripotency genes at the end of Stage 3 were increased compared to the original protocol under normoxic conditions (Fig. S1F, G). More importantly, we observed significant CiPSC colonies (Fig. 1D, S1H, I) and eventually established multiple CiPSC lines from the same ADSC sample across different batches. All these CiPSCs exhibited the typical morphology of PSCs, characterized by flat colonies, tight cell-cell contact, and a high nuclear-to-cytoplasmic ratio (Fig. S1J). Immunofluorescence analysis further confirmed that these CiPSCs expressed the core pluripotency markers OCT4, NANOG, SOX2 and SSEA4 (Fig. S1L, Table S3), and high-resolution G-banding karyotype analysis revealed that CiPSC lines maintained a normal karyotype (Fig. S1M).

Meanwhile, we also generated TF-based iPSC lines from the same ADSC sample via electroporation of three plasmids carrying the coding sequences for SOX2, KLF4, OCT3/4, L-MYC, and LIN28A (Liu et al. 2021). These cells displayed the typical morphology of PSCs (Fig. S1K), expressed core pluripotent markers (Fig. S1L) and exhibited normal karyotypes (Fig. S1M), with no exogenous plasmid DNA residue detected by PCR analysis (Fig. S1N). To evaluate the isogenic iPSCs and CiPSCs, we first performed an RT-qPCR experiment showing that the expression levels of pluripotency genes between CiPSC and iPSC lines were highly similar and comparable (Fig. S1O, Table S4). Moreover, the RNA-seq-based transcriptomic analysis indicated that CiPSCs and iPSCs exhibited consistent gene expression patterns, with high levels of pluripotency-related genes and low levels of lineage-specific differentiation genes (Fig. S1P). No significant differences were observed in cell cycle profiles or population doubling times between the two cell types (Fig. S1Q, R). Teratoma assays indicated that both were able to form teratomas in immunodeficient mice containing structures representative of all three germ layers (ectoderm, mesoderm, and endoderm), confirming their tri-lineage differentiation potential (Fig. S1S). Taken together, these CiPSC and iPSC lines exhibited a high degree of consistency of pluripotency.

Human umbilical cord-derived mesenchymal stem/stromal cells (UC-MSCs) are one of the most widely used somatic cell types in translational and clinical applications, due to their robust proliferation capacity, low immunogenicity, and abundant banking resources (Mebarki et al. 2021). Therefore, we attempted to reprogram human UC-MSCs into CiPSCs, utilizing a similar strategy previously applied for ADSC reprogramming (Table S1-S2). Distinct from the ADSC case, lower concentrations of small molecules were more beneficial for UC-MSCs (Fig. S2A). Similarly, both DFOM and ITS-X together with B27/G27 could enhance the expression of *LIN28A* at the end of Stage 1 (Fig. 1E, S2B). In addition, given that cell reprogramming involves massive epigenetic changes (Wen et al. 2025), we tested several chemicals targeting histone modifications, and found that the KAT3A/KAT3B catalytic inhibitor SGC-CBP30 enhanced *LIN28A* expression (Fig. S2C), a result consistent with the recent report of the rapid CiPSC platform that the KAT3 serves as a key epigenetic obstacle to reprogramming (Wang et al. 2025). We thus evaluated A-485, another KAT3 inhibitor used in the same study, and observed a similar promotive effect on *LIN28A* expression during Stage 1 (Fig. S2D). These optimizations together constitute our improved protocol for UC-MSC reprogramming.

We next monitored the reprogramming process of UC-MSCs under normoxic conditions. Our improved protocol significantly increased *LIN28A* expression, and the expression levels of stage-specific markers across the other two stages were determined by RT-qPCR (Fig. 1F-H). Immunofluorescence analysis indicated an increase in LIN28A-positive cells (Fig. 1I, S2E). Consistently, flow cytometry analysis further confirmed that our protocol significantly improved the percentage of LIN28A-positive cells (Fig. 1J). Notably, the cell morphology appeared more regular and reached high confluence in a shorter time, up to 10 days (Fig. S2F). Moreover, our protocol induced a more pronounced multi-layered cell accumulation structure at Stage 2 with a shorter time (Fig. S2H), along with a significant increase in the number of SALL4-positive cells (Fig. 1K, L, S2G). Consistent with the above observations, our protocol yielded a markedly higher number of CiPSC colonies at Stage 3 (Fig. 1M, N, S2I), with positive expression of core pluripotency markers (Fig. S2J). Finally, we established several CiPSC lines from UC-MSCs. All these CiPSCs exhibited the typical morphology of PSCs (Fig. S2K) and presented a population doubling time comparable to that of ESC line H9 (Fig. 1O). Immunofluorescence and RT-qPCR analyses confirmed the expression of pluripotency markers in the CiPSCs (Fig. 1P, Q). RNA-seq analysis further revealed that CiPSCs and the ESC line H9 shared comparable transcriptional profiles, suggesting that CiPSCs had successfully transitioned from a somatic to a pluripotent state (Fig. 1R, S2L). Additionally, the teratoma assay confirmed the tri-lineage differentiation potential (Fig. 1S), and high-resolution G-banding karyotype analysis confirmed that CiPSCs maintained a normal karyotype (Fig. 1T).

To validate the robustness of our improved protocol, we applied it to other batches of UC-MSCs from different donors (01-UC-MSC, 03-UC-MSC). Consistently, CiPSC colonies emerged at Stage 3 of reprogramming and RT-qPCR analysis revealed a significant upregulation of pluripotency-associated genes (Fig. S2M-O). Moreover, CiPSC lines established from different UC-MSC donors were positive for core pluripotency markers (Fig. S2P).

In this study, we generated human CiPSCs from ADSCs and UC-MSCs through tailored optimization of chemical reprogramming protocols. We noticed that a recent preprint also reported the establishment of CiPSCs from frozen human umbilical cord tissue based on the original “four-stage” system (Guan et al. 2022) (10.1101/2024.04.10.588154v1). The successful induction of stable CiPSC lines from multiple UC-MSC donors, together, further supports the robustness and adaptability of chemical reprogramming across distinct somatic cell types. In addition, side-by-side comparisons between isogenic CiPSCs and TF-iPSCs indicated broadly comparable pluripotency and lineage-differentiation potential between the two reprogramming methods.

Notably, our optimization results suggest that modulation of core small-molecule conditions and KAT3-associated epigenetic barriers plays an important role in efficient UC-MSC reprogramming, whereas DFOM and ITS-X mainly served supportive roles during early-stage induction and epithelial-like cell generation. Interestingly, compared with ADSCs, lower concentrations of several small molecules were effective enough for Stage 1 induction in UC-MSCs. This may reflect the relatively anti-inflammatory intrinsic transcriptional and inflammatory profile of UC-MSCs (Ganguly et al. 2023), whereas JNK-associated pro-inflammatory signaling has been identified as a key barrier to human chemical reprogramming (Guan et al. 2022). In addition, distinct epigenetic landscapes associated with different tissue origins and cell types may further contribute to differential sensitivities to small-molecule induction.

Collectively, these findings support the reproducibility and robustness of chemical reprogramming and provide an optimized strategy for generating human CiPSCs from ADSCs and UC-MSCs under normoxic conditions. Nevertheless, the current protocol still relies on research-grade reagents such as FBS and Matrigel and further optimization of chemically defined and GMP-grade culture conditions will be required to facilitate future translational applications.

## Supplementary Information

Supplementary Material 1. Supplementary Methods and Figures. Fig. S1: Chemical reprogramming of human ADSCs into CiPSCs. Fig. S2: Chemical reprogramming of human UC-MSCs into CiPSCs.

Supplementary Material 2. Supplementary Tables. Table S1: Reprogramming medium. Table S2: Chemicals information. Table S3: Antibody information. Table S4: RT-qPCR primers information.

## Data Availability

RNA sequencing (RNA-seq) data have been deposited in the Genome Sequence Archive (GSA) under​ accession number HRA018371.

## Abbreviations

UC-MSC: Umbilical cord-derived mesenchymal stem/stromal cell
ADSC: Adipose-derived stromal cell
CiPSC: Chemically induced pluripotent stem cell
iPSC: Induced pluripotent stem cell
PSC: Pluripotent stem cell
ESC: Embryonic stem cell
DFOM: Deferoxamine mesylate
PCR: Polymerase chain reaction
RT-qPCR: Reverse transcription-quantitative polymerase chain reaction
RNA-seq: RNA sequencing
