# Supplementary material for: Generation of human chemically induced pluripotent stem cells from UC-MSCs
Source: Cell Regen. 2026 Jul 1;15:20. doi: 10.1186/s13619-026-00295-9 (PMC13319313; doi:10.1186/s13619-026-00295-9)
Supplement: Supplementary file 1 — Supplementary Material 1. Supplementary Methods and Figures. Fig. S1: Chemical reprogramming of human ADSCs into CiPSCs. Fig. S2: Chemical reprogramming of human UC-MSCs into CiPSCs. [file 13619_2026_295_MOESM1_ESM.docx]

**Generation of human chemically induced pluripotent stem cells from UC-MSCs**

Guangqiang Gong^1,#^, Shanshan Wen^1,#^, Siyi Huang^1^, Ran Liu^2^, Ran Zheng^2,4^*, Wei Jiang^1,2,3,^*

Supplementary Methods

Supplementary Figures:

Fig. S1: Chemical reprogramming of human ADSCs into CiPSCs.

Fig. S2: Chemical reprogramming of human UC-MSCs into CiPSCs.

**Supplementary Methods**

**Animals**

All animal experiments in this study were approved by the Wuhan University Committee for the Management and Use of Laboratory Animals. Five-week-old male BALB/c nude immunodeficient mice purchased from Vital River were used for teratoma assay. Mice were housed in SPF-level facilities under the unified management of the Wuhan University Medical Research Institute Laboratory Animal Center, maintained under a 12-hour light-dark cycle. Water and food were accessible at all times.

**Cell culture**

Human ESC H9, and generated iPSC and CiPSC lines were cultured on Matrigel-coated plates in mTeSR1 medium (STEMCELL), with daily medium changes. Human ADSCs were cultured in DMEM media (Gibco) containing 15% Fetal Bovine Serum (FBS, Gibco) and 1% penicillin-streptomycin (PS, Gibco), with medium changes every two days. Human UC-MSCs were cultured in human umbilical cord mesenchymal stem/stromal cell serum-free complete medium (OriCell), with medium changes every two days. Occasionally, human ADSCs could be also cultured in UC-MSC culture medium. All cells were cultured in an incubator at 37°C with 5% CO₂.

Human ADSCs were isolated from one breast adipose tissue sample provided by the collaborative laboratory. Human UC-MSCs (Wharton's jelly source) used in this study were obtained from Beike Biotechnology. Jiang laboratory’s work on human ESCs and iPSCs is approved by the Biomedical Ethics Committee of Wuhan University (WHU-LFMD-IRB2024026). All procedures were conducted in accordance with the International Society for Stem Cell Research (ISSCR) Guidelines for Stem Cell Research and Clinical Translation and complied with the Helsinki Declaration of 1975 (as revised in 2008).

**Generation of CiPSCs by chemical reprogramming**

The entire reprogramming process was conducted under 21% O_2_. Human ADSCs and UC-MSCs were seeded at densities of 1×10⁴ cells per well in 12-well plates, using Knockout DMEM (Gibco) supplemented with 15% FBS, 1% GlutaMAX (Gibco), 1% nonessential amino acids (NEAA, Gibco), and 1% PS.

For ADSC reprogramming, except for the modifications listed below, the composition of the culture media for each stage is consistent with the previously reported three-stage protocol (Liuyang et al., 2023). The Stage 1 medium optimization is as follows: adjusted concentrations of key small molecules CHIR99021, RepSox, TTNPB, VTP50469, JNK-IN-8, and EPZ5676 to 10 μM, 20 μM, 4 μM, 1 μM, 0.4 μM, and 4 μM, respectively; 4% B27, 1% ITS-X and 2-4 μM DFOM were added. The Stage 2 medium optimization is as follows: 4% B27 and 2 μM RA were added. The reprogramming was initiated on the following day and the Stage 1 medium was added and lasted approximately 8-10 days, during which the epithelial-like cells achieve approximately 100% confluence in the monolayer. Subsequently, the Stage 2 medium was applied for approximately 8-10 days. Finally, the three sub-stages for Stage 3 lasted for 4, 4, and 2-4 days, respectively.

For UC-MSC reprogramming, the optimization for Stage 1 medium is as follows: adjusted concentrations of key small molecules CHIR99021, RepSox, TTNPB, VTP50469, JNK-IN-8, EPZ5676, and DZNep to 2.5 μM, 5 μM, 1 μM, 0.25 μM, 0.1 μM, 1 μM, and 0.025 μM, respectively; 1% ITS-X and 2-4 μM DFOM were added; and G27 (bioGenous) was used as a substitute to B27. The reprogramming was initiated on the following day. The Stage 1 lasted for approximately 8-10 days (with an optional 1-2 days extension), during which the epithelial-like cells achieved approximately 100% confluence in the monolayer with **moderate piling**. Stage 2 lasted for 6-10 days, and the three sub-stages for Stage 3 lasted for 2-3, 2-3, and 2-4 days, respectively. The media formula for each stage was provided in Table S1, and the information about the chemicals used was listed in Table S2.

**Derivation and culture of CiPSC lines**

After 6-10 days of induction under the Stage 3 conditions, the cells were dissociated using Accutase, centrifuged at 300g for 3 minutes, and replated at a split ratio ranging from 1:3 to 1:8 onto Matrigel-coated **plates**. The cells were maintained in the cell line derivation medium (Wang et al., 2025). After 10-12 days, compact CiPSC colonies were mechanically dissected into small clumps and transferred onto Matrigel-coated plates in mTeSR1 medium containing Y-27632 (10 µM). After 24 hours, the medium was replaced with fresh mTeSR1 medium.

**Generation of iPSCs by overexpressing transcription factors**

Human ADSCs were electroporated with three episomal plasmids (Addgene #27076, #27078, and #27080) using the Human Dermal Fibroblasts Nucleofector Kit (LONZA) on the LONZA 4D instrument and cultured with ADSC medium containing Y-27632 (10 µM). Y-27632 could be withdrawn around 6 to 24 hours after electroporation. 5-7 days later, the medium was changed into a 1:1 mixture of ADSC medium and ReproTeSR medium (STEMCELL) for 2 days, followed with complete ReproTeSR medium for 7 to 12 days, a 1:1 mixture of ReproTeSR medium and mTeSR1 medium for 2 days, and complete mTeSR1 medium. Approximately 1 to 2 weeks after electroporation, iPSC colonies may be observed. The cells could be dissociated using Accutase and re-seeded at a 1:4 ratio if the cell density becomes too high.

**Immunofluorescence**

Cells cultured in 24-well plates were washed with DPBS (Gibco), followed by fixation with 4% paraformaldehyde (PFA, DING GUO) for 20 minutes at room temperature. After washing, the cells were blocked and permeabilized in a solution containing 10% donkey serum and 0.3% Triton X-100 in DPBS for 2 hours. Subsequently, the cells were incubated with primary antibodies diluted in the blocking solution at 4°C overnight or at room temperature for 2 hours. Following three washes with DPBS (each for 5-10 minutes on a rocking shaker), the cells were incubated with fluorophore-conjugated secondary antibodies for 2 hours at room temperature in the dark. After another three washes with DPBS, the cells were counterstained with 4,6-diamidino-2-phenylindole (DAPI, Sigma) diluted in DPBS for 10 minutes at room temperature in the dark. Fluorescent images were acquired using an OLYMPUS IX53 inverted fluorescence microscope. To quantify positive visual fields, three random visual fields per sample were uniformly analyzed in ImageJ using the same threshold criteria. The antibodies used in this study were listed in Table S3.

**Flow cytometry**

The cells were washed with DPBS and dissociated with Accutase at 37°C. Then the cells were suspended in DPBS with 2% FBS and collected by centrifuging at 1000 rpm for 3 minutes at 4°C. Subsequently, the cells were fixed and permeabilized using Fix/Perm Buffer (BD Biosciences) for 1 hour at 4°C. Following this, the cells were washed twice with Perm Buffer (BD Biosciences) and incubated with primary antibodies overnight at 4°C. After washing, the cells were incubated with fluorophore-conjugated secondary antibodies for 3 hours at 4°C in the dark. Thereafter, the cells were washed and resuspended in 200 μL DPBS. Finally, the cells were analyzed using a flow cytometer (Beckman Cytoflex LX) and data were analyzed using FlowJo software.

**Reverse Transcription-Quantitative Polymerase Chain Reaction Analysis**

Following DPBS wash and lysis in Buffer RL, total RNA was isolated from harvested cells using the HiPure Total RNA Mini Kit (Magen) as per the manufacturer’s instructions. The extracted RNA was quantified on a NanoDrop 2000 spectrophotometer (Thermo Fisher), and reverse transcription was carried out with the ABScript II RT Master Mix (ABclonal) to generate cDNA. Quantitative RT-PCR analysis based on the ΔCt method was conducted in a 384-well format on a C1000 Touch Thermal Cycler (Bio-Rad) using 2× Universal SYBR Green Fast qPCR Mix (ABclonal). Gene expression levels were normalized to *GAPDH*, which served as an internal control. The primer sequences used in this study were listed in Table S4.

**Population doubling time**

The cell growth rate was determined by periodically counting cell numbers using a hemocytometer. Data points from the exponential growth phase were used for analysis. The population doubling time (DT) was calculated according to the following formula: **DT = t × [ln2 / (ln*N_t_* – ln*N_0_*)]**, where N_0_ represents the initial cell number at time zero, and N_t_ represents the cell number at a later time point t.

**Cell cycle analysis**

For cell cycle analysis, cells were fixed overnight in 70% ethanol at 4°C. The following day, cells were washed with DPBS and treated with RNase A at 37°C for 30 minutes to remove RNA. After washing, the cells were stained with Propidium Iodide (PI) in the dark for 30 minutes. The stained cells were washed twice, resuspended in DPBS, and then **detected** using a flow cytometer (Beckman Cytoflex LX). Cell cycle distribution (G1, S, G2/M phases) was analyzed using FlowJo software.

**Teratoma assay**

For teratoma formation, cells were dissociated with Accutase, resuspended in mTeSR1 medium, and counted. Approximately 2×10⁶ cells were collected, mixed with 60 μL Matrigel, and injected into the hind limbs of 2-to-3-month-old immune-deficient mice. After 24 hours, mouse survival was confirmed, and the animals were transferred to new cages, with subsequent health status monitoring every two weeks. Approximately 7 weeks post-injection, teratomas had formed and the mice were euthanized. The teratomas were surgically harvested from the injection site, washed with DPBS, and fixed in 4% PFA for 24 hours. The fixed samples were then processed for paraffin embedding and hematoxylin and eosin staining.

**Karyotype analysis**

The karyotype (chromosomal G-band) analyses were performed by Zhongnan Hospital of Wuhan University using standard protocol for high-resolution G-banding (400G–500G). The results were analyzed by CytoVision (Leica). For each analysis, the number of chromosomes as well as the presence of structural chromosomal abnormalities of at least 20 metaphases were examined.

**RNA sequencing (RNA-seq) and Data analysis**

Total RNA was extracted using the HiPure Total RNA Mini Kit for RNA sequencing. Following sequencing on the DNBSEQ platform, the raw reads were processed with fastp (v0.24.0) for quality control. The clean reads were then aligned to the human genome build hg38/GRCh38 using HISAT2 (v2.2.1), and raw counts were ultimately quantified with FeatureCounts (v2.0.8).

**Statistical analysis**

Statistical analyses were performed using GraphPad Prism 10. *P* values were calculated using one-way ANOVA followed by Dunnett’s test in Figure 1A, B and Supplementary Figure 2A, D; one-way ANOVA followed by Tukey’s test in Supplementary Figure 2B; and unpaired two-tailed Student’s t-test for the remaining comparisons. Data are represented as mean ± SD, with statistical significance defined as **p* < 0.05, ***p* < 0.01, ****p* < 0.001. The number of biological replicates is indicated in the corresponding figure legends.

**References**

Liuyang, S., Wang, G., Wang, Y., He, H., Lyu, Y., Cheng, L.,…Deng, H. (2023). Highly efficient and rapid generation of human pluripotent stem cells by chemical reprogramming. *Cell Stem Cell*, *30*(4), 450-459.e459. <https://doi.org/10.1016/j.stem.2023.02.008>

Wang, Y., Peng, F., Yang, Z., Cheng, L., Cao, J., Fu, X.,…Deng, H. (2025). A rapid chemical reprogramming system to generate human pluripotent stem cells. *Nature Chemical Biology*, *21*(7), 1030-1038. <https://doi.org/10.1038/s41589-024-01799-8>

**Supplementary Figures**


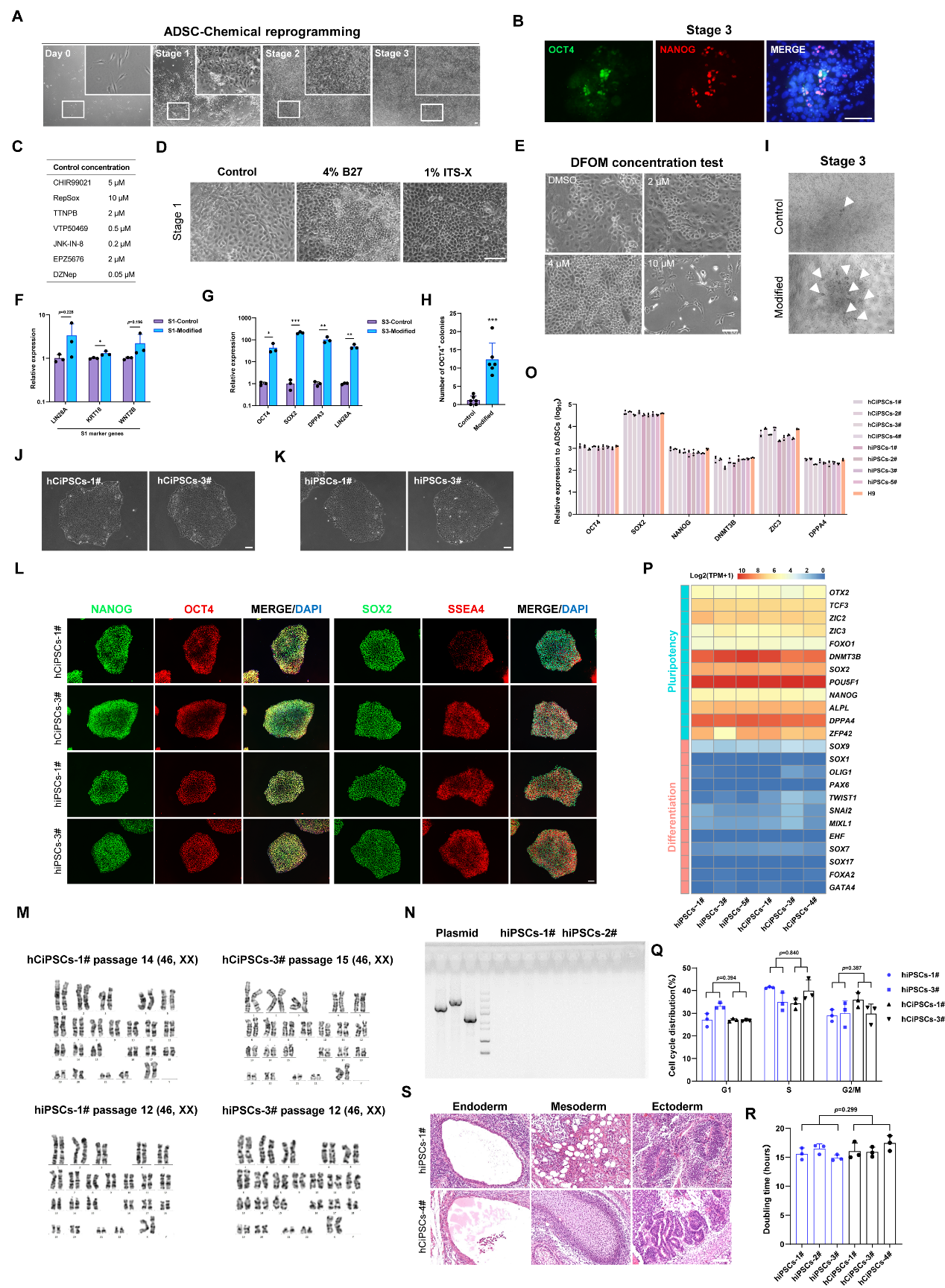


**Fig. S1:** **Chemical reprogramming of human ADSCs into CiPSCs.**

**A**. Representative images of cells at the end of each stage during CiPSCs induction from ADSCs under the original protocol. Scale bar, 100 μm.

**B**. Immunofluorescence of pluripotency markers in primary CiPSCs from ADSCs at the end of Stage 3 under the original protocol. Scale bar, 100 μm.

**C**. Concentrations of the seven core small molecules applied during Stage 1 under the original protocol.

**D.** Cell morphology at the end of Stage 1 following supplementation with 4% B27 or 1% ITS-X under the original protocol. Scale bar, 100 μm.

**E.** Cell morphology at the end of Stage 1 following supplementation with different concentrations of DFOM under the original protocol. Scale bar, 100 μm.

**F**. RT-qPCR analysis of somatic and first-stage marker genes at the end of Stage 1 under the original protocol (control) and modified protocol. Data represented as mean ± SD (n = 3).

**G**. RT-qPCR analysis of pluripotency marker genes at the end of Stage 3 under the original protocol (control) and modified protocol. Data represented as mean ± SD (n = 3).

**H**. Number of CiPSC colonies at the end of Stage 3 under the original protocol (control) and modified protocol. Data represented as mean ± SD (one well of a 24-well plate counted as one group, n = 6).

**I**. Cell morphology at the end of Stage 3 under the original protocol (control) and modified protocol. Arrows indicate CiPSC colonies. Scale bar, 100 μm.

**J-K.** Morphology of CiPSCs (J) and iPSCs (K) from the same donor. Scale bar, 100 μm.

**L**. Immunofluorescence of pluripotency markers in isogenic CiPSCs and iPSCs from the same donor. Scale bar, 100 μm.

**M.** Karyotype analysis showing a normal diploid chromosome content in CiPSCs and iPSCs from the same donor.

**N.** PCR detection of plasmid backbone sequences in iPSCs derived from ADSCs. Lanes from left to right: positive control plasmids (pCXLE-hUL, pCXLE-hSK, pCXLE-hOCT3/4), DNA marker, and iPSCs.

**O**. RT-qPCR analysis of pluripotency marker genes in isogenic CiPSCs and iPSCs from the same donor. ESC H9 was used as a reference. Data represented as mean ± SD (n = 3).

**P**. Heatmaps showing the expression of pluripotency- and differentiation-related genes in CiPSCs and iPSCs.

**Q.** Cell cycle analysis in these isogenic CiPSCs and iPSCs from the same donor. Data represented as mean ± SD. For iPSC and CiPSC, data from three biological replicates per clone were averaged for statistical analysis (n = 2 clones).

**R.** Calculated doubling times for isogenic CiPSCs and iPSCs from the same donor. Data represented as mean ± SD. For iPSC and CiPSC, data from three biological replicates per clone were averaged for statistical analysis (n = 3 clones).

**S**. Hematoxylin and eosin (H&E) staining of teratoma sections from the same donor, showing CiPSCs and iPSCs. For each CiPSC and iPSC line, images contained tissues representative of endoderm, mesoderm, and ectoderm from the same teratoma. Scale bar, 100 μm.

**
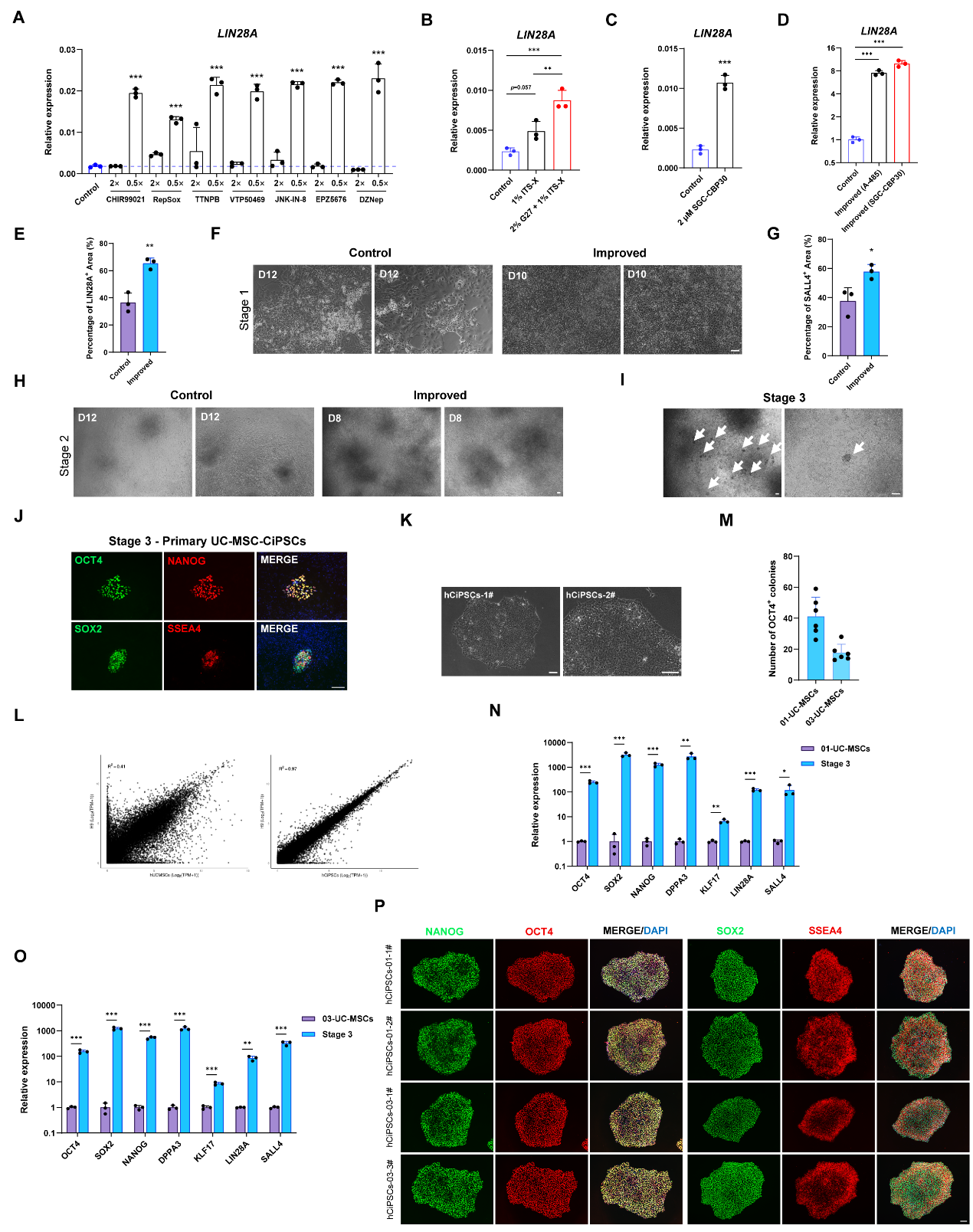
**

**Fig. S2: Chemical reprogramming of human UC-MSCs into CiPSCs**.

**A.** RT-qPCR analysis of *LIN28A* at the end of Stage 1 upon concentration titrations of core small molecules under the original protocol (control). Data represented as mean ± SD (n = 3).

**B.** RT-qPCR analysis of *LIN28A* at the end of Stage 1 following supplementation with 1% ITS-X or 1% ITS-X + 2% G27 under the original protocol (control). G27 was used as a replacement for B27. Data represented as mean ± SD (n = 3).

**C.** RT-qPCR analysis of *LIN28A* at the end of Stage 1 following supplementation with 2 μM SGC-CBP30 under the original protocol (control). Data represented as mean ± SD (n = 3).

**D.** RT-qPCR analysis of *LIN28A* at the end of Stage 1 under the original protocol (control) and improved protocols (treated with the KAT3 inhibitors A-485 and SGC-CBP30, respectively). Data represented as mean ± SD (n = 3).

**E.** Percentage of LIN28A-positive area at the end of Stage 1 under the original protocol (control) and improved protocol. Three random visual fields were analyzed for each sample. Data represented as mean ± SD.

**F.** Cell morphology at the end of Stage 1 under the original protocol (control) and improved protocol. Scale bar, 100 μm.

**G.** Percentage of SALL4-positive area at the end of Stage 2 under the original protocol (control) and improved protocol. Three random visual fields were analyzed for each sample. Data represented as mean ± SD.

**H.** Cell morphology at the end of Stage 2 under the original protocol (control) and improved protocol. Scale bar, 100 μm.

**I.** Cell morphology at the end of Stage 3 under the improved protocol. Arrows indicate CiPSC colonies from UC-MSCs. Scale bar, 100 μm.

**J.** Immunofluorescence of pluripotency markers in primary CiPSCs from UC-MSCs at the end of Stage 3 under the improved protocol. Scale bar, 100 μm.

**K.** Morphology of CiPSCs from UC-MSCs. Scale bar, 100 μm.

**L.** Scatter plot comparing global gene expression profiles of CiPSCs, ESC H9, and UC-MSCs.

**M.** Number of CiPSC colonies from 01-UC-MSCs and 03-UC-MSCs at the end of Stage 3 under the improved protocol. Data represented as mean ± SD (n = 6, one well of a 24-well plate was counted as one group).

**N**. RT-qPCR analysis of pluripotency marker genes at the end of Stage 3 of 01-UC-MSC reprogramming under the improved protocol. Data represented as mean ± SD (n = 3).

**O.** RT-qPCR analysis of pluripotency marker genes at the end of Stage 3 of 03-UC-MSC reprogramming under the improved protocol. Data represented as mean ± SD (n = 3).

**P.** Immunofluorescence of pluripotency markers in CiPSCs from 01-UC-MSCs and 03-UC-MSCs. Scale bar, 100 μm.
